# Effect of CO_2_-induced seawater acidification on growth, photosynthesis and inorganic carbon acquisition of the harmful bloom-forming marine microalga, *Karenia mikimotoi*

**DOI:** 10.1371/journal.pone.0183289

**Published:** 2017-08-16

**Authors:** Shunxin Hu, Bin Zhou, You Wang, Ying Wang, Xinxin Zhang, Yan Zhao, Xinyu Zhao, Xuexi Tang

**Affiliations:** Department of Marine Ecology, College of Marine Life Sciences, Ocean University of China, Qingdao, China; University of Connecticut, UNITED STATES

## Abstract

*Karenia mikimotoi* is a widespread, toxic and non-calcifying dinoflagellate, which can release and produce ichthyotoxins and hemolytic toxins affecting the food web within the area of its bloom. Shifts in the physiological characteristics of *K*. *mikimotoi* due to CO_2_-induced seawater acidification could alter the occurrence, severity and impacts of harmful algal blooms (HABs). Here, we investigated the effects of elevated *p*CO_2_ on the physiology of *K*. *mikimotoi*. Using semi-continuous cultures under controlled laboratory conditions, growth, photosynthesis and inorganic carbon acquisition were determined over 4–6 week incubations at ambient (390ppmv) and elevated *p*CO_2_ levels (1000 *ppmv* and 2000 *ppmv*). pH-drift and inhibitor-experiments suggested that *K*. *mikimotoi* was capable of acquiring HCO_3_^-^, and that the utilization of HCO_3_^-^ was predominantly mediated by anion-exchange proteins, but that HCO_3_^-^ dehydration catalyzed by external carbonic anhydrase (CA_ext_) only played a minor role in *K*. *mikimotoi*. Even though down-regulated CO_2_ concentrating mechanisms (CCMs) and enhanced gross photosynthetic O_2_ evolution were observed under 1000 ppmv CO_2_ conditions, the saved energy did not stimulate growth of *K*. *mikimotoi* under 1000 ppmv CO_2_, probably due to the increased dark respiration. However, significantly higher growth and photosynthesis [in terms of photosynthetic oxygen evolution, effective quantum Yield (Yield), photosynthetic efficiency (α), light saturation point (E_k_) and ribulose-1,5-bisphosphate carboxylase/oxygenase (Rubisco) activity] were observed under 2000 ppmv CO_2_ conditions. Furthermore, elevated *p*CO_2_ increased the photo-inhibition rate of photosystem II (β) and non-photochemical quenching (NPQ) at high light. We suggest that the energy saved through the down-regulation of CCMs might lead to the additional light stress and photo-damage. Therefore, the response of this species to elevated CO_2_ conditions will be determined by more than regulation and efficiency of CCMs.

## Introduction

Ocean acidification refers to the ongoing reduction in the ocean pH over an extended period of time, which is primarily caused by the uptake of anthropogenic CO_2_ from the atmosphere [1, 2]. Industrialization and fossil fuel combustion have increased the atmospheric CO_2_ concentrations from pre-industrial levels of approximately 280 *ppmv* to the current level of approximately 390 *ppmv* [2, 3]. The atmospheric CO_2_ concentrations are predicted to increase to 1000 *ppmv* and 2000 *ppmv* by the years 2100 and 2300, respectively, if the present energy utilization structure persists [1]. Such increases in CO_2_ would lead to a reduction in pH (0.4 and 0.77 units, respectively) and cause substantial chemical changes in seawater carbonate systems, including increases in *p*CO_2_, HCO_3_^-^ and DIC and decreases in H^+^ and CO_3_^2-^ [4, 5].

Marine phytoplankton assimilates inorganic carbon and fixes CO_2_ into carbohydrates through the enzyme ribulose-1,5-bisphosphate carboxylase/oxygenase (Rubisco), which can only use CO_2_ as substrate for the carboxylase reaction. Rubisco is generally characterized by low affinities for CO_2_ (*K*_M_ of 20–70 μmol L^-1^), and a competitive reaction with O_2_ further reduces its efficiency [6]. Therefore, photosynthesis of some marine phytoplankton might suffer from CO_2_ limitation, due to the present concentration of aqueous CO_2_ in seawater ranging from 8 to 20 μmol L^-1^. Most marine phytoplankton have developed so-called CO_2_ concentrating mechanisms (CCMs) to overcome the deficiencies of Rubisco. Two main types of CCMs are suggested to be involved in marine phytoplankton. Firstly, the dehydration of HCO_3_^-^ is catalyzed by external carbonic anhydrase, facilitating the supply of CO_2_ at plasma membrane and improving the potential for CO_2_ uptake, and then CO_2_ accumulation is achieved by the active transport of HCO_3_^-^ or CO_2_ at the chloroplast envelope. Secondly, HCO_3_^-^ is transported across the plasmalemma and/or chloroplast envelope and then converted to CO_2_, catalyzed by one of several types of internal carbonic anhydrase [7]. However, the expression and operation of CCMs require energetic investment. Therefore, marine phytoplankton could benefit from elevated *p*CO_2_ in two synergetic ways: one is that the increased dissolved carbon dioxide (CO_2_aq) would supply additional substrate for photosynthetic carbon fixation and reduce the oxygenase reaction of Rubisco, alleviating the carbon limitations of species without a CCM [8]. The other is that, increased *p*CO_2_ could down-regulate the energetically costly operation of CCMs.

Many marine phytoplankton species down-regulate their operation of CCMs at high *p*CO_2_ conditions, as revealed by a lower photosynthetic affinity for CO_2_, decreased activities of carbonic anhydrase and/or a lower contribution of HCO_3_^-^assimilation [9–12]. This is taken as evidence that elevated *p*CO_2_ exerts positive effects on the growth and photosynthesis of species bearing CCMs[9, 13–15]. Some species (*Macrocystis pyrifera* and *Gracilaria lemaneiformis*), however, do not show any deactivation of CCMs or changes in growth and photosynthesis in response to elevated *p*CO_2_ [16, 17]. In addition, increased *p*CO_2_ might exert negative effects on calcifying species because of a lowering of saturation of CaCO_3_, which might make calcfication more difficult [18,19]. Deleterious effects of elevated *p*CO_2_, however, also occur on non-calcifying species [20–23], probably due to the negative effects on physiological processes caused by reduced external pH. [24]. Altogether, these findings havedeepened our understandings of differential physiological responses to elevated *p*CO_2_ among various marine phytoplankton species, such as diatoms and coccolithophores.

*Karenia mikimotoi* is a widespread, toxic and non-calcifying dinoflagellate, which can have deleterious effects on other marine phytoplankton, fish and shellfish through the release of ichthyotoxins and hemolytic toxins [25]. *K*. *mikimotoi* toxic blooms have been reported in Japan, Ireland, England, France, India and China [26–30]. At least 103 harmful algal blooms (HABs) have been reported between 2004 and 2014 caused by *K*. *mikimotoi* in eastern and southern Chinese seas, affecting about 37527 km^2^ (according to China Marine Disaster Bulletin). Owing to its ecological and environmental implications, it is necessary to study the physiological responses of this species to elevated *p*CO_2_ in order to predict the occurrence, severity and impacts of blooms of this species in the future. In the present study, we evaluated growth, photosynthesis, dark respiration and the CCMs modes of *K*. *mikimotoi* exposed to three different *p*CO_2_ levels: 390 *ppmv* (pH_NBS_: 8.10) which is the present pH value, as well as 1000 *ppmv* (pH_NBS_: 7.78) and *p*CO_2_: 2000 *ppmv* (pH_NBS_: 7.49), which are predicted to be possible conditions in 2100 and 2300, respectively. We hypothesized that (1) the operation of CCMs will be down-regulated with elevated *p*CO_2_ and (2) the reduction in the energy costs of CCMs will benefit growth and photosynthesis of *K*. *mikimotoi*.

## Materials and methods

### Culture conditions and experimental design

*Karenia mikimotoi* (strain OUC151001) was obtained from the Algal Culture Collection at the Ocean University of China. Cells were cultured in 0.45μm-filtered natural seawater, collected from Luxun Seaside Park (Qingdao), which had been autoclaved (30min, 121°C) and enriched with f/2 medium [31]. All cultures were incubated at 20±1°C and illuminated with 80 μmol photon m^-2^ s^-1^ under a 12:12 light: dark cycle.

Experiments were conducted in triplicate 1000 ml sterilized and acid-washed Erlenmeyer flask containing 600 ml of medium. Prior to inoculation, the cultures were equilibrated at three different CO_2_ levels: 390 *ppmv* CO_2_ (~present-day), 1000 and 2000 *ppmv* CO_2_ (predicted CO_2_ levels in 2100 and 2300, respectively), obtained by gentle bubbling with 0.22 μm-filtered ambient air and air/CO_2_ mixtures. The air/CO_2_ mixtures were generated by plant CO_2_ chambers (HP400G-D, Ruihua Instrument & Equipment Ltd, Wuhan, China) with a variation of less than 5%. Semi-continuous cultures were used to measure the effects of CO_2_-induced seawater acidification on the growth and physiology of *K*. *mikimotoi* in the present study, similar to previous ocean acidification research [9, 32–34]. All cultures were diluted to 800 cells mL^-1^ with fresh medium pre-acclimated to the desired CO_2_ level every 24h to maintain cells in exponential growth phase, and to minimize pH fluctuations. Cultures were harvested following 4–6 weeks of semi-continuous incubation when the growth rates were not significantly different for three or more consecutive days, which was considered fully acclimated to their respective experimental treatments.

### Seawater carbonate chemistry

The pH value and dissolved inorganic carbon (DIC) were determined prior to and after the daily dilution. The concentration of DIC in the culture medium was measured using a total organic carbon analyzer (TOC-V_CPN_, Shimadzu). The samples were filtered onto brown glasses via 0.45 μm cellulose acetate membranes and stored in a refrigerator (4°C). The pH was measured using a pH meter (SevenCompact^™^ S210k, Mettler Toledo, Switzerland), which was calibrated daily with standard National Bureau of Standards (NBS) buffer system. The other relevant parameters of carbonate system were determined with the CO_2_SYS software [35], based on the known parameters (pH, DIC, salinity and temperature).

### Growth and elemental analysis

Cell growth rate was monitored daily using a plankton counting chamber (0.1 mL) before and after the medium was diluted. The specific growth rate (μ) was calculated using the equation:

$$\mu =\left(\text{ln}N_{1}-\text{ln}N_{0}\right)/\left(t_{1}-t_{0}\right),$$

where N_0_ and N_1_ represent the average cell numbers at times t_0_ (after the dilution) and t_1_ (before the dilution), respectively.

Samples for measurements of cellular carbon (C), nitrogen (N) and phosphorus (P) were filtered onto glass microfiber membranes (GF/F, Whatman), which were pre-combusted at 500°C for 4 h, and then stored at -20°C in a refrigerator before analysis. Cellular C and N content were determined using a Perkin-Elmer 2400 CHNS analyzer following the method of Zhao et al. [36]. Cellular P content was measured as in Fourqurean et al. [37].

### Chlorophyll *a*

Chlorophyll *a* (Chl *a*) samples from the cultures were filtered onto glass microfiber filters (GF/F, Whatman), and extracted with 10 mL of methanol overnight at 4°C. Samples were then analyzed in a spectrophotomer, and the Chl *a* concentration was calculated according to the following equation [38]:

$$\text{Chl a}=16.29\times \left(A_{665}-A_{750}\right)-8.54\times \left(A_{652}-A_{750}\right)$$

where A_652,_ A_665_ and A_750_ denoted the absorbance values of the methanol extracts at 652nm, 665nm and 750nm, respectively.

### Photosynthetic oxygen evolution and respiration

Photosynthetic oxygen evolution was measured under the same light intensities used for growing cultures, with lighting provided by a halogen lamp. The dark respiration rate was determined using a Clark-type oxygen electrode (Chlorolab 3, Hansatech, UK). Experimental temperature was maintained at 20°C using a water bath circulator. Before the determination, cells were acclimated to light or dark conditions in the reaction chamber for 20 min. The 5ml-reaction media was continuously stirred with a magnetic stirrer during treatment. DIC concentrations were consistent with their culture conditions, which were nominally 390ppmv: 1919–1924 μmol/kg; 1000ppmv: 2059–2067 μmol/kg; 2000 ppmv:2152–2156 μmol/kg, respectively.

### Chlorophyll fluorescence measurements

Fluorescence induction curves and rapid light curves (RLCs) were applied to evaluate the photosynthetic performance of *K*. *mikimotoi* acclimated to different *p*CO_2_, using a Water-PAM fluorometer.

The RLCs were measured at 8 different actinic irradiance levels (80, 119, 184, 276, 393, 546, 897 and 1315 μmol photon m^-2^ s^-1^), each of which lasted 10s. To quantitatively compare the RLCs of *K*. *mikimotoi* acclimated to different *p*CO_2_, the equation of Platt et al. [39] was applied to derive characteristic parameters: photosynthetic efficiency (α), light saturation point (E_k_), photo-inhibition rate of photosystemII(β) and maximum relative electron transport rate (rETR_max_). The light saturation point was determined from: *E*_*k*_ = *rETR*_*max*_/α [40]. Related parameters were applied to determine the convergence of the regression mode according to Ralph and Gademann [40].

For the fluorescence induction curves, all of the samples were dark-acclimated for 20 mins before determination. The dark-acclimation induction curves were measured with a delay of 40 s between the determinations of F_v_ / F_m_. The actinic light was set at 80, 276 and 897 μmol photon m^-2^ s^-1^, respectively, to measure the value of effective quantum yield (Yield) and non-photochemical quenching (NPQ)

### Determination of ribulose-1,5-bisphosphate carboxylase/oxygenase (Rubisco) activities

Cells were collected by centrifugation at a rotating speed of 3000g at 4°C for 15 min. After removing the supernatant, cells were grinded on ice with the addition of 1mL buffer solution (40 mM Tris-HCl, 5 mM Glutathione, 10 mM MgCl_2_ and 0.25 mM EDTA, pH 7.6). The liquid was concentrated, and the supernatant was used for further assays. Rubisco activity in the supernatant was generally determined following the methods described by Gerard and Driscoll [41]. The assay mixture contained 5 mM NADH, 50 mM ATP, 50 mM phosphocreatine, 0.2 mM NaHCO_3_, 160 U/mL creatine phosphokinase, 160 U/mL Phosphoglycerate kinase, 160 U/mL glyceraldehyde-3-phosphate dehydrogenase and reaction buffer (0.1M Tris-HCl, 12 mM MgCl_2_ and 0.4 mM EDTA, pH 7.8). Absorbance values at 340 nm (A_340_) were measured every 20s for 3 min to obtain the background NADH oxidation rate. A volume of 0.05 mL of RuBP (final concentrations of 25 mM) was added into the assay mixture, and the A_340_ was then recorded every 20 s for 3 min. The activities of Rubisco were computed by subtracting the background rate of decrease in A_340_ from the rate determined during the three minutes following RuBP addition, and then converting the corrected rate of A_340_ decrease to a rate of NADH oxidation.

### pH drift experiment

A pH drift experiment was applied to determine whether *K*. *mikimotoi* can utilize HCO_3_^-^ as an inorganic carbon source; the ability of algae to raise the medium pH to higher than 9.0 is considered as evidence of its capacity to utilize HCO_3_^-^. The experiment was performed in sterilized glasses containing 10 mL samples (cell concentrations of 10×10^4^ mL^-1^) at 20°C and 80 μmol photon m^-2^ s^-1^. The pH was measured every hour and the final pH values were obtained once no further pH increases were detected.

### Determination of carbonic anhydrase activity

Cells were collected by centrifugation at a rotating speed of 3000g at 4°C for 15 min and re-suspended in 20mM barbitone (pH 8.2). The total carbonic anhydrase (CA_tot_) and external carbonic anhydrase (CA_ext_) activities were measured using an electrometric method [42]. For determination of CA_tot_, cells were disrupted with a sonicator, and cell brakage confirmed under a microscope. The reaction was begun by adding 2 mL ice-cold CO_2_ to saturated Milli-Q water, and the time it took for the pH to decrease from 8.2 to 7.2 was recorded. The temperature was controlled at 4°C.

### Effect of inhibitors on chlorophyll fluorescence

The RLCs of *K*. *mikimotoi* acclimated to different *p*CO_2_ with addition of inhibitors were obtained to determine the mechanism of inorganic carbon acquisition. The inhibitors included acetazolamide (AZ), which inhibits only extracellular CA, ethoxyzolamide (EZ), which inhibits both extracellular and intracellular CA, and DIDS (4,4’-diisothiocyanostilbene-2,2’-disulfonate), which inhibits direct HCO_3_^-^ uptake by means of the anion-exchange protein. These inhibitors have been widely used to determine the contribution of external CA, internal CA and anion-exchange protein to photosynthetic inorganic carbon uptake [21, 43, 44].

### Statistical analysis

One-way ANOVA was used to analyze the significance of the differences between treatments using the SPSS software (20.0), and the significant difference level was set to *P* < 0.05. All figures were prepared with Sigmaplot 12.5.

## Results

### Seawater carbonate chemistry

Under the simulated laboratory conditions of ocean acidification, the seawater carbonates chemistry system at elevated *p*CO_2_ (1000 *ppmv* and 2000 *ppmv*) levels significantly differed from that of the control group (Table 1). The DIC, CO_2_ and HCO_3_^-^ concentrations in the 1000 *ppmv*-treated system were increased by 7.0%, 125.9% and 10.1%, respectively, whereas in the 2000 *ppmv*-treated system, these values increased by 12.1%, 361.4% and 15.5%, respectively. The CO_3_^2-^ concentrations were decreased by 46.5% and 71.1% in the 1000 *ppmv*- and 2000 *ppmv*-treated systems, respectively, and the difference in total alkalinity (TA) was insignificant. The fluctuations of pH prior and after the dilution of the culture medium were <0.03.

**Table 1:** Parameters of the seawater carbonate chemistry system at different *p*CO_2_ levels prior and after the dilution. The dissolved inorganic carbon (DIC) concentration, pH_NBS_, temperature and salinity were used to compute other parameters with a CO_2_ system analyzing software (CO_2_SYS). Data are shown as the mean ± SE (n = 9). Different letters represent significant difference between variables (P < 0.05).

		pH_NBS_	DIC/(μmol/kg)	HCO_3_^-^/(μmol/kg)	CO_3_^2-^/(μmol/kg)	CO_2_/(μmol/kg)	TA/(μmol/kg)
Control	Prior	8.13±0.02^a^	1919.6±9.0 ^a^	1762.8±4.0 ^a^	142.3±5.4 ^a^	14.4±1.4 ^a^	2113.6±16.9 ^a^
	After	8.10±0.02^a^	1924.6±14.7 ^a^	1773.9±12.6 ^a^	135.2±2.3 ^a^	15.4±1.2 ^a^	2110.9±17.3 ^a^
1000*ppmv*	Prior	7.81±0.01^b^	2067.7±14.3^b^	1957.7±12.6 ^b^	75.5±2.4 ^b^	33.6±0.6 ^b^	2143.9±18.0 ^a^
	After	7.78±0.02 ^b^	2059.6±14.2 ^b^	1951.6±12.7 ^b^	72.4±2.0 ^b^	34.8±0.5 ^b^	2131.9±17.3 ^a^
2000*ppmv*	Prior	7.52±0.01^c^	2152.6±11.6 ^c^	2053.3±24.0 ^c^	41.2±1.3 ^c^	67.7±0.8 ^c^	2145.9±13.1 ^a^
	After	7.50±0.02 ^c^	2156.3±14.6 ^c^	2048.7±9.8 ^c^	39.1±1.3 ^c^	71.0±1.6 ^c^	2141.1±14.0 ^a^

### Growth and elemental composition

**Fig 1:**
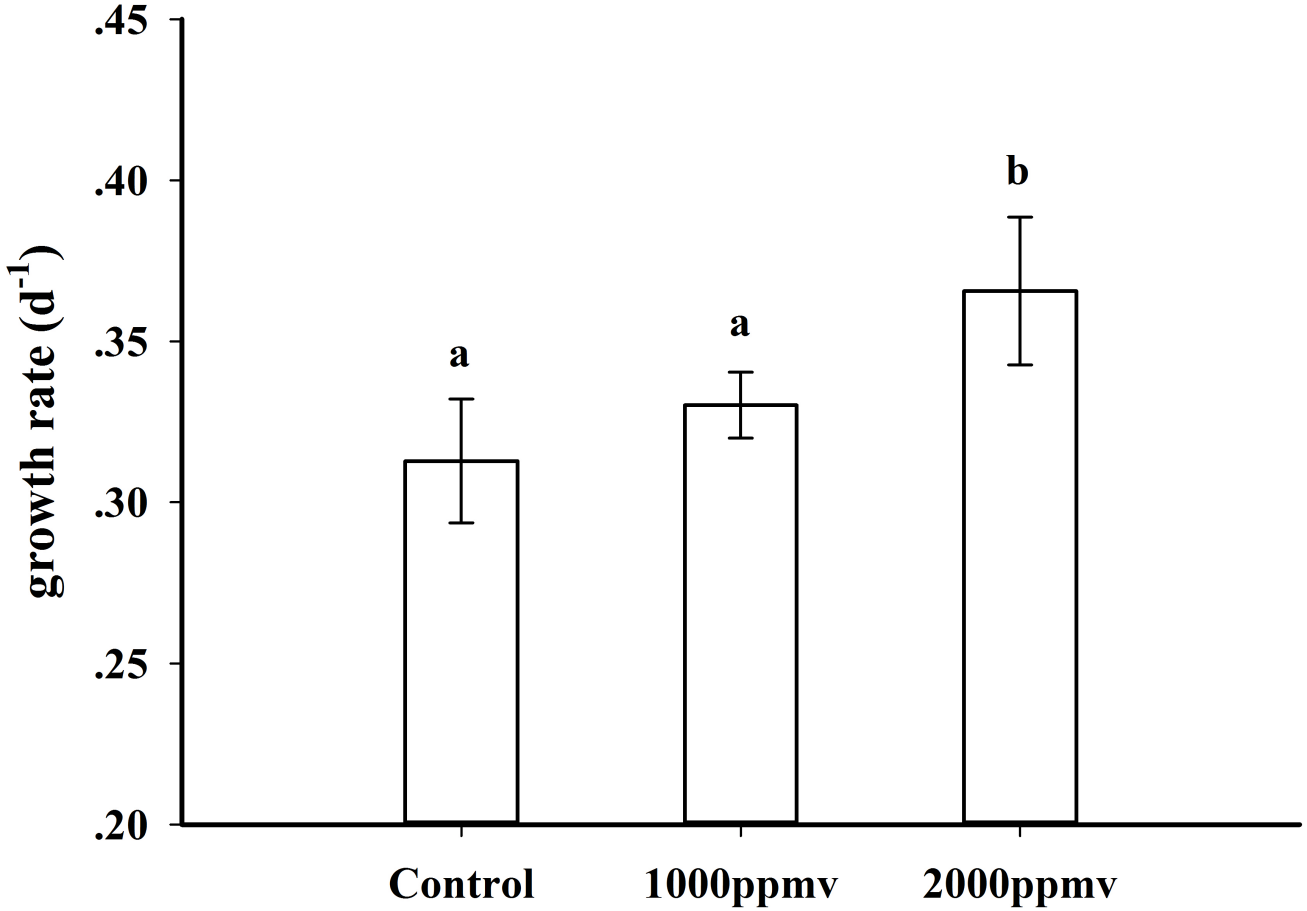
Growth rate of *K*. *mikimotoi* acclimated to different *p*CO_2_ levels. Data are shown as the mean ± SE (n = 9).

The growth rates at the three *p*CO_2_ levels are shown in Fig 1. The growth rate of *K*. *mikimotoi* was significantly stimulated by 16.84% (P<0.05) after exposure to 2000 *ppmv p*CO_2_; although growth was also enhanced under 1000 *ppmv p*CO_2_, the increase was not statistically significant (P>0.05).

The total cellular C,N,P and their elemental ratio in *K*. *mikimotoi* are shown in Table 2. The cellular C and P concentrations of *K*. *mikimotoi* exposed to 2000 *ppmv p*CO_2_ levels were significantly (P<0.05) higher than those of the control, whereas there was no significant difference between the control and 1000 *ppmv p*CO_2_ (P>0.05). Furthermore, elevated *p*CO_2_ exerted no significant effects on the cellular N, C: N, C: P and N: P ratios (P>0.05).

**Table 2:** Elemental composition (total C, N and P) and elemental ratio (C:N, C:P and N:P) of *K*. *mikimotoi* acclimated to different *p*CO_2_ levels. Data are shown as the mean ± SE (n = 9). Different letters represent significant difference between variables (P < 0.05).

*p*CO_2_	C pmol cell^-1^	N pmol cell^-1^	P pmol cell^-1^	C:N	C:P	N:P
Control	6.43±0.70^a^	1.23±0.07^a^	0.082±0.007^a^	5.27±0.85^a^	78.2±2.8^a^	15.09±2.36^a^
1000 *ppmv*	7.63±0.46^ab^	1.33±0.03 ^a^	0.081±0.004 ^a^	5.72±0.23^a^	93.9±9.9^a^	16.39±1.14^a^
2000 *ppmv*	7.99±0.36^b^	1.34±0.11 ^a^	0.093±0.003^b^	5.96±0.32^a^	85.2±7.2^a^	14.29±0.92^a^

### Chlorophyll *a*

**Fig 2:**
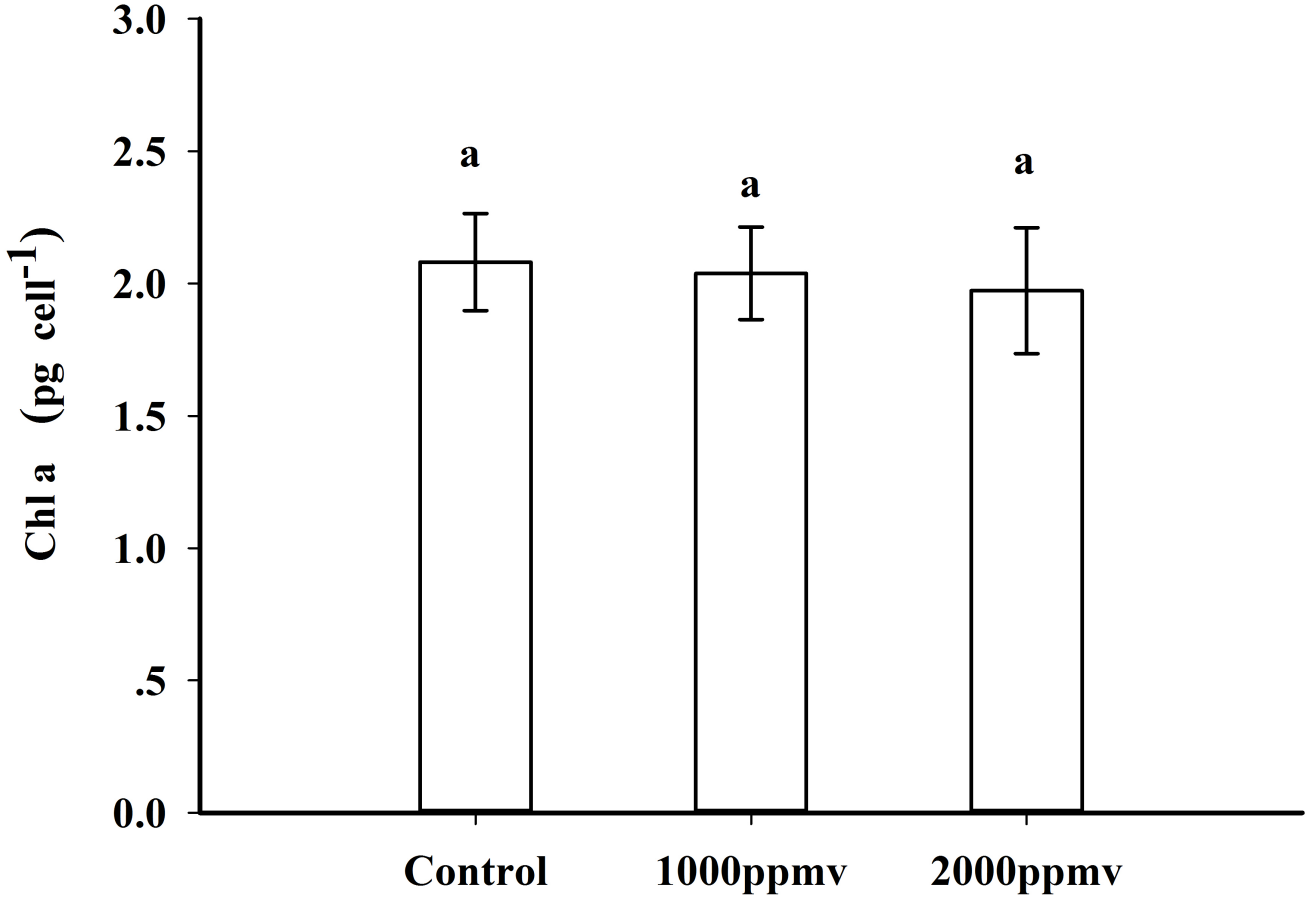
Chlorophyll *a* content of *K*. *mikimotoi* acclimated to different *p*CO_2_ levels. Data are shown as the mean ± SE (n = 9).

Cells acclimated to the three *p*CO_2_ levels showed the same Chl *a* content (Fig 2) of about 2.0 pg cell^-1^, with no significant differences among treatments.

### Photosynthetic oxygen evolution, dark respiration and Rubisco activities

**Fig 3:**
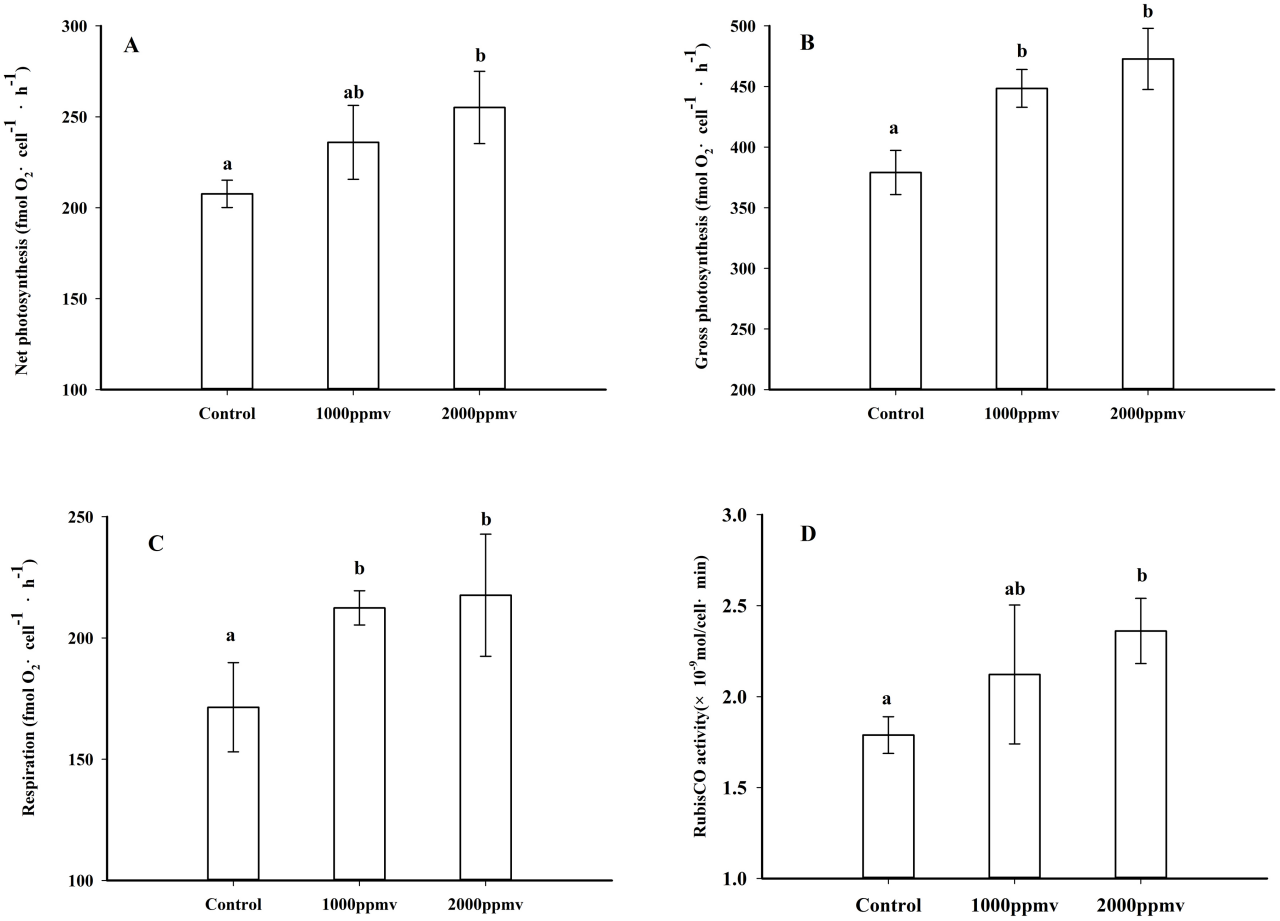
Net photosynthetic oxygen evolution (A), gross photosynthetic oxygen evolution (B), dark respiration (C) and Rubisco activity (D) of *K*. *mikimotoi* acclimated to different *p*CO_2_ levels. Data are shown as the mean ± SE (n = 9).

Results of the determinations of the net photosynthetic oxygen evolution, gross photosynthetic oxygen evolution, dark respiration, and Rubisco activity are shown in Fig 3. Net photosynthetic oxygen evolution (Fig 3A) and Rubisco activity (Fig 3D) were significantly enhanced by 22.86% (P<0.05) and 31.99% (P<0.05) under 2000 *ppmv p*CO_2_, whereas there were no significant difference between the control and 1000 *ppmv p*CO_2_ (P>0.05). Cells acclimated to both 1000 *ppmv* and 2000 *ppmv p*CO_2_ treatments showed higher gross photosynthetic oxygen evolution (Fig 3B) and dark respiration (Fig 3C) than those of the control (P<0.05).

### Chlorophyll fluorescence

**Fig 4:**
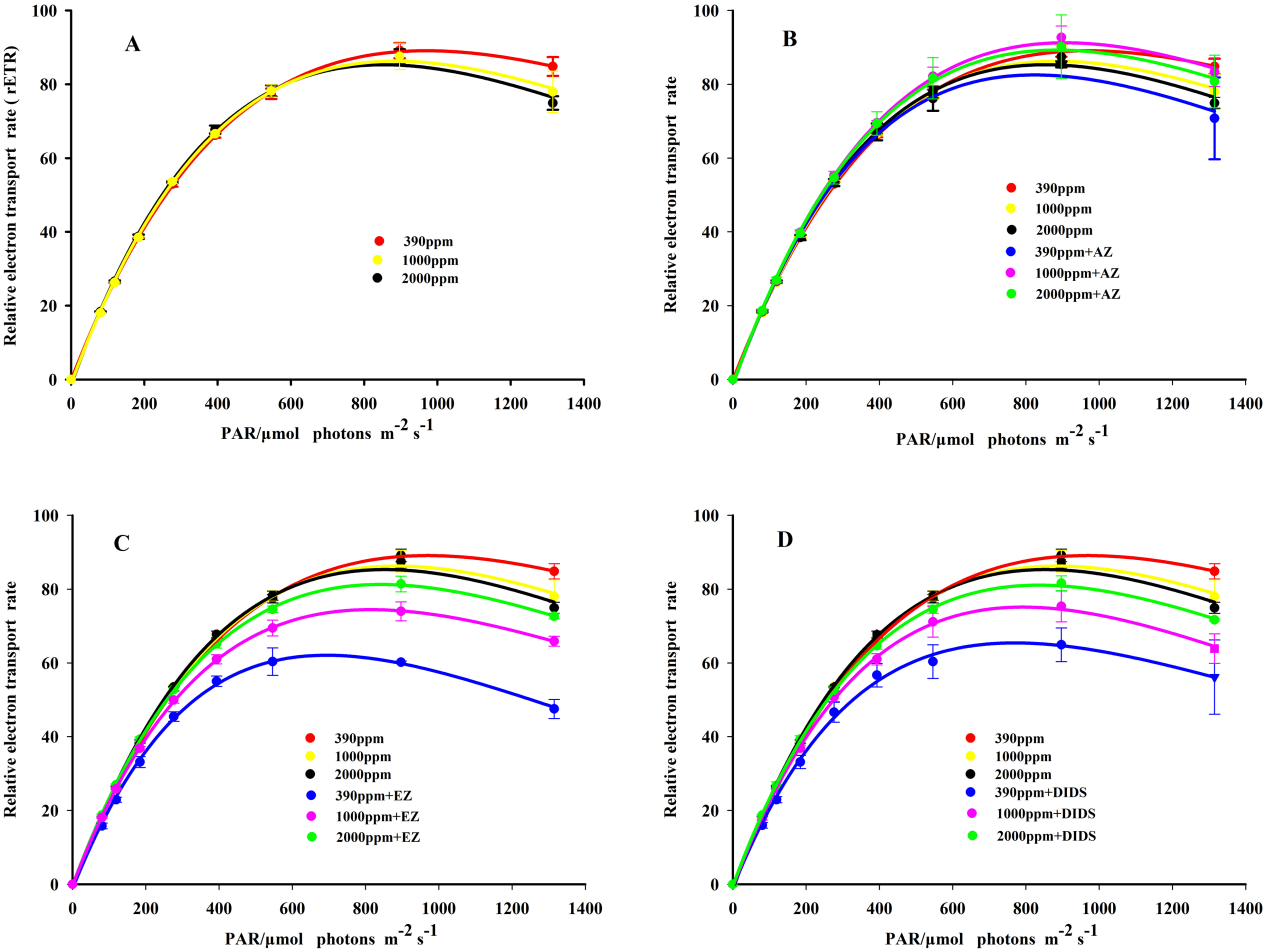
The rapid light curves of *K*. *mikimotoi* without and with the addition of inhibitors (AZ, EZ and DIDS) acclimated to different *p*CO_2_. Data are shown as the mean ± SE (n = 3).

Rapid light curves (RLCs) were determined at the three levels of *p*CO_2_ (control, 1000 *ppmv* and 2000 *ppmv*). The three treatments all exhibited a classical pattern of rETR as a function of PAR (Fig 4A), with a rapid increase under light-limited conditions followed by a plateau, at which the photosynthetic pathway was saturated.

The parameters derived from the RLCs are shown in Table 3. The photosynthetic efficiency (α) of *K*. *mikimotoi* was significantly stimulated by 23.1% (P<0.05) under 2000 *ppmv p*CO_2._ Cells acclimated to 1000 *ppmv p*CO_2_ also showed higher α than that of control, but the increase was not statistically significant (P>0.05). Contrary to the trend observed in α, elevated *p*CO_2_ significantly decreased the light saturation point (E_k_) of *K*. *mikimotoi* by 7.5% (P<0.05) and 10.2% (P<0.05) under 1000 and 2000 *ppmv* CO_2_ compared with the control, respectively. The maximum relative electron transport rates (rETR_max_) were approximately 10% higher in both elevated *p*CO_2_ levels compared with the control, but the differences were not significant (P > 0.05). Moreover, the results showed that increased *p*CO_2_ levels had no significant effect on the F_v_ / F_m_ of *K*. *mikimotoi* (P > 0.05).

**Table 3:** Photosynthetic parameters derived from the rapid light curves of *K*. *mikimotoi* acclimated to different *p*CO_2_ levels. Data are shown as the mean ± SE (n = 3). Different letters represent significant difference between variables (P < 0.05).

*p*CO_2_	Fv/Fm	A	rETR_max_	E_k_	β
**390 ppmv**	0.599±0.007^a^	0.238±0.0150^a^	82.8±5.2^a^	347.6±5.8^a^	0.048±0.007^a^
**1000 ppmv**	0.603±0.008^a^	0.285±0.0289^a^	91.5±5.5^a^	315.7±6.6^b^	0.110±0.027^b^
**2000 ppmv**	0.605±0.007^a^	0.293±0.0256^b^	91.6±10.9^a^	312.2±14.9^b^	0.143±0.012^b^

The inhibition of rETR was obtained from the rapid light curves in all three different *p*CO_2_ levels when exposed to the actinic irradiance of 1315 μmol photon m^-2^ s^-1^. β values (relative inhibition), characterizing the photo-inhibition rate of PSII exposed to high actinic irradiance, were significantly increased by 126.7% (P<0.01) and 194.3% (P<0.001), respectively, in 1000 *ppmv* and 2000 *ppmv p*CO_2_ compared with the control (Table 3).

**Fig 5:**
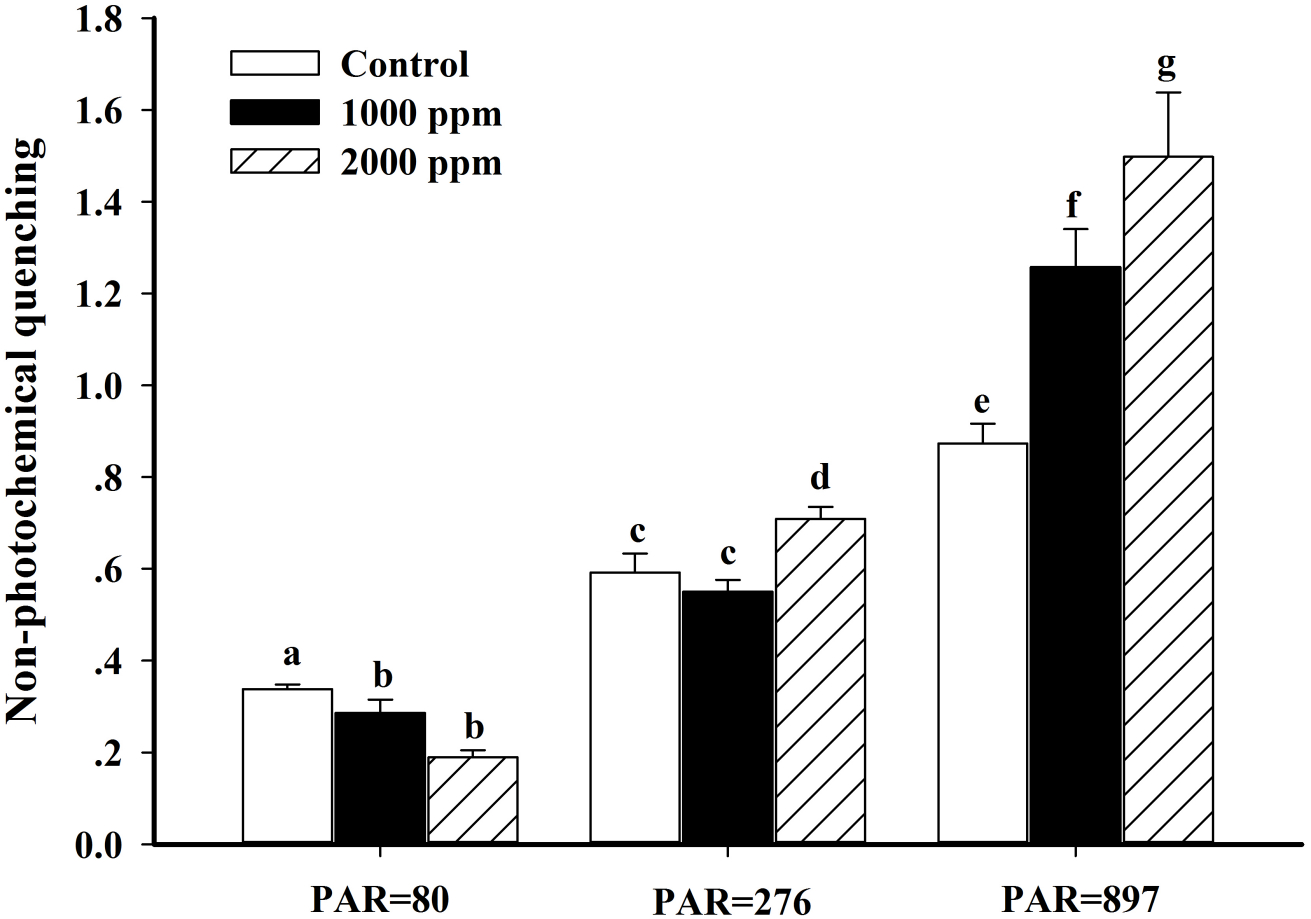
Non-photochemical quenching (NPQ) of *K*. *mikimotoi* acclimated to different *p*CO_2_ levels at an actinic irradiance of 80, 276 and 897 photon m^-2^ s^-1^. Data are shown as the mean ± SE (n = 3).

**Fig 6:**
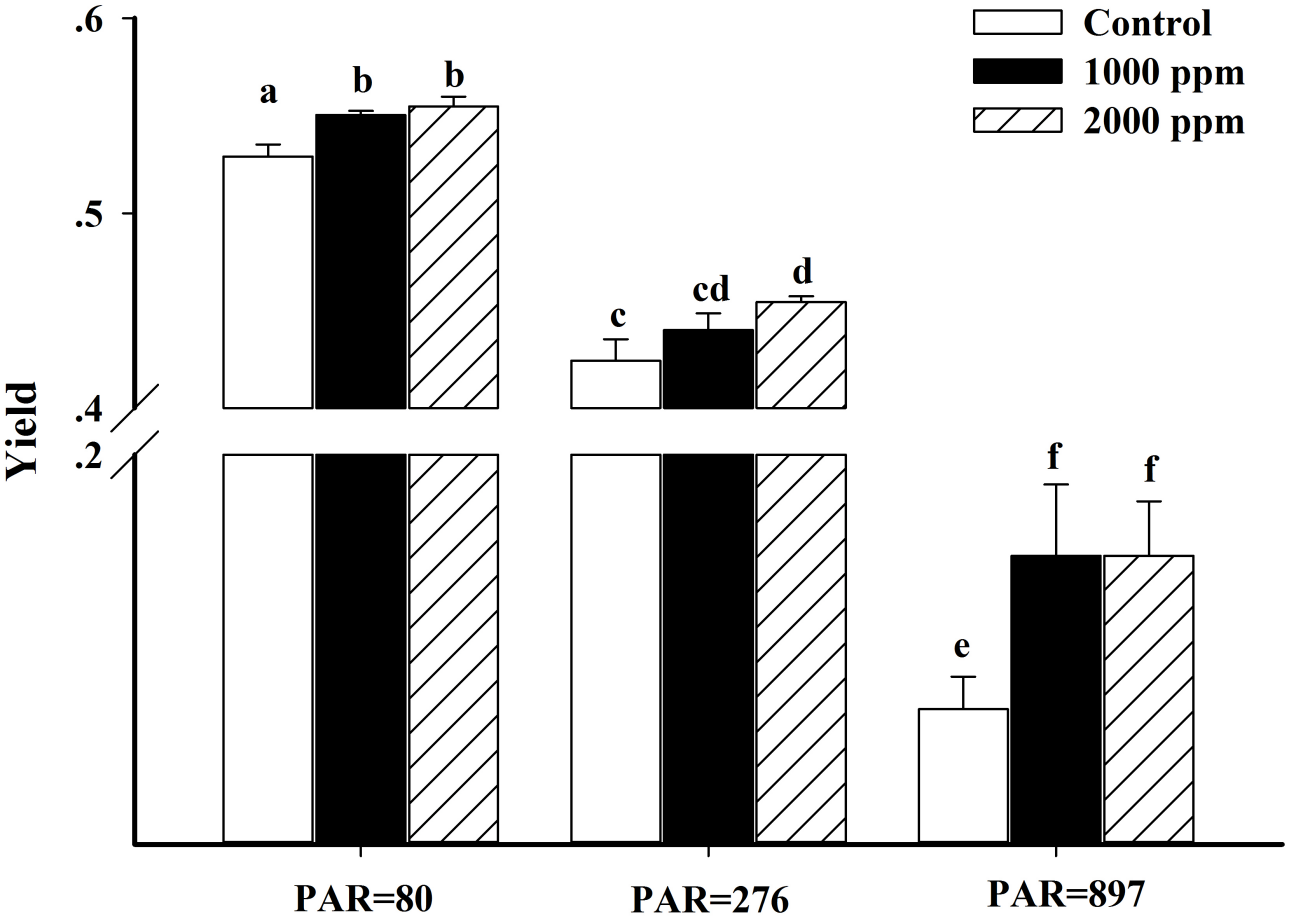
Effective quantum yield (Yield) of *K*. *mikimotoi* acclimated to different *p*CO_2_ levels at an actinic irradiance 80, 276 and 897 photon m^-2^ s^-1^. Data are shown as the mean ± SE (n = 3).

The non-photochemical quenching (NPQ) and effective quantum yield of PS II (Yield) values derived from the induction light curve (IC) are shown in Figs 5 and 6, respectively. The results indicated that response of NPQ to higher CO_2_ concentrations depended on actinic irradiances. Under 80 μmol photon m^-2^ s^-1^, the NPQ values in the 390*ppmv p*CO_2_ were 15.4% (P<0.05) and 43.9% (P<0.01) higher than those observed in the 1000 *ppmv* and 2000 *ppmv p*CO_2_, respectively. By contrast, NPQ values at 276 and 897 μmol photon m^-2^ s^-1^ were significantly increased in the two high *p*CO_2_ groups (*P* < 0.01), but there was no significant difference between 1000 *ppmv* and the control when exposed to 276 μmol photon m^-2^ s^-1^ (P>0.05). Relative to the control conditions, both the 1000 *ppmv* and 2000 *ppmv p*CO_2_ groups significantly stimulated the Yield, which was increased by 4.0% (P<0.05) and 4.9% (P<0.01) at 80 μmol photon m^-2^ s^-1^, and increased by 11.8% (P<0.05) and 11.8% (P<0.05) at 897 μmol photon m^-2^ s^-1^.

At an actinic of 276 μmol photon m^-2^ s^-1^, the 2000 *ppmv p*CO_2_ groups exhibited a significant (P<0.01) increase, but not the 1000 *ppmv p*CO_2_ groups.

### pH drift experiment and carbonic anhydrase activity

**Fig 7:**
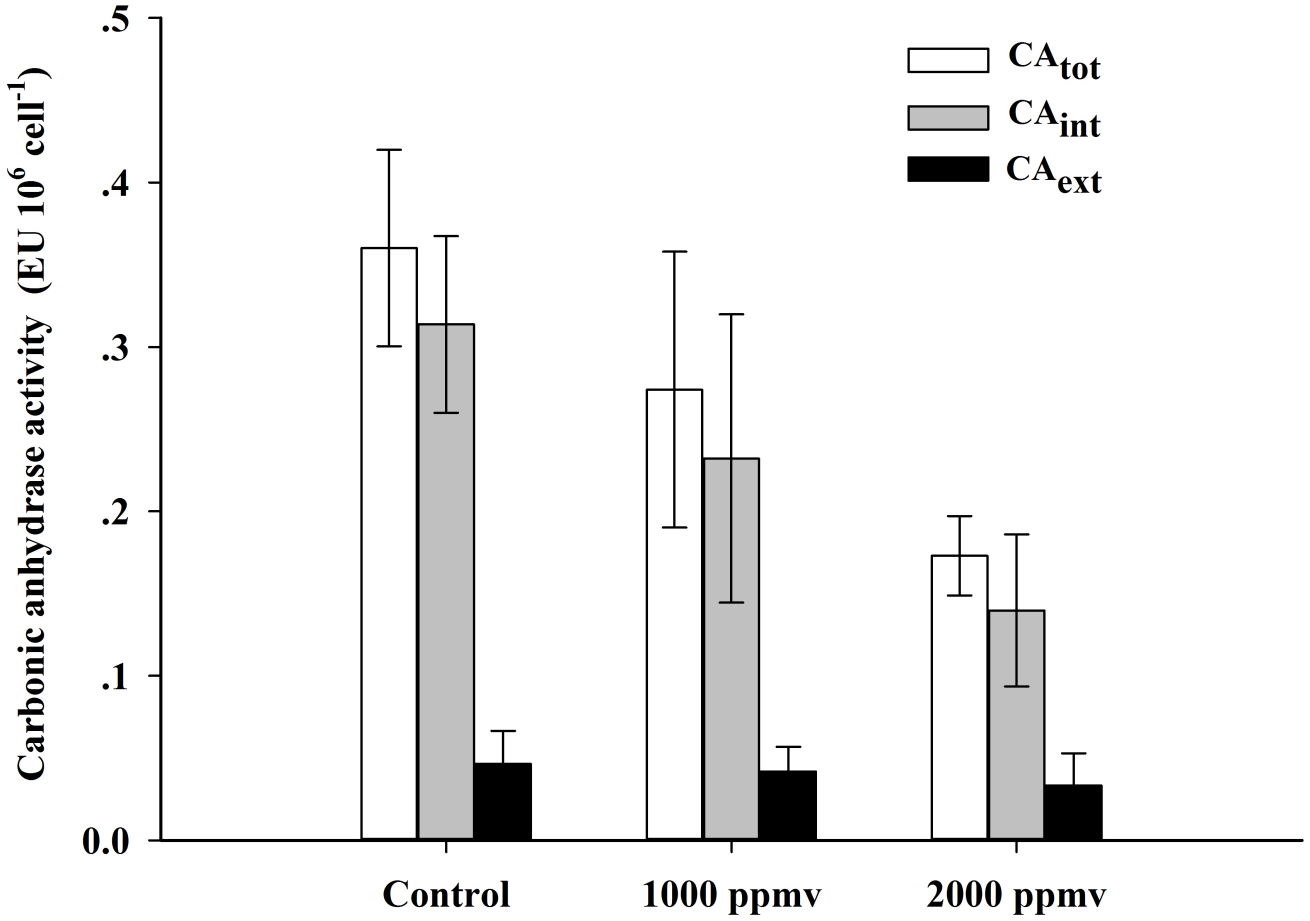
Total, external and internal carbonic anhydrase activity of *K*. *mikimotoi* acclimated to different *p*CO_2_ levels. Data are shown as the mean ± SE (n = 3).

The final pH value obtained in the pH drift experiment was 9.8±0.1. Furthermore, the total carbonic anhydrase activity (CA_tot_) and internal anhydrase activity (CA_int_) (Fig 7) of *K*. *mikimotoi* were significantly decreased by 50.6% (P<0.01) and 55.5% (P<0.05) after exposure to 2000 *ppmv p*CO_2_, and no significant changes were observed when exposed to 1000 *ppmv p*CO_2_ (P>0.05). The external carbonic anhydrase activity (CA_ext_) (Fig 7) of *K*. *mikimotoi* was significantly lower than the CA_int_, and no significant changes were observed when exposed to 1000 *ppmv* (P>0.05) and 2000 *ppmv* (P>0.05) *p*CO_2_.

### Effect of inhibitors on chlorophyll fluorescence

The rapid light curves of *K*. *mikimotoi* acclimated to different *p*CO_2_ levels with and without the addition of AZ, EZ and DIDS are shown in Fig 4. The results indicated that the rETR values of *K*. *mikimotoi* were significantly inhibited by the addition of EZ and DIDS, whereas the inhibition of rETR by the addition of AZ was significantly less than that obtained with EZ and DIDS.

The inhibition rates of rETR at different *p*CO_2_ levels obtained with the addition of AZ, EZ and DIDS are shown in Table 4. The results indicated that the inhibitions of rETRs by the addition of EZ and DIDS were significantly (P<0.01) higher in the controls than those obtained at the high *p*CO_2_ groups (1000 *ppmv* and 2000 *ppmv*). Furthermore, the results from all three *p*CO_2_ levels indicated that the inhibition of rETR was significantly increased by the increasing PAR. in the presence of AZ, rETR inhibition was only observed in the 390 *ppmv p*CO_2_.

**Table 4:** Percent inhibition of rETR acclimated to different *p*CO_2_ with the addition of AZ, EZ and DIDS within a PAR range of 0 to 1315 μmol photon m^-2^ s^-1^, “—” represents no inhibition of rETR. Data are shown as the mean ± SE (n = 3).

PAR μmol photon m^-2^s^-1^	Inhibition rate of rETR								
	390*ppmv*+AZ	1000*ppmv*+AZ	2000*ppmv*+AZ	390*ppmv*+EZ	1000*ppmv*+EZ	2000*ppmv*+EZ	390*ppmv*+DIDS	1000*ppmv*+DIDS	2000*ppmv*+DIDS
**80**	—	—	—	13.04±5.44%	—	—	13.06±4.42%	1.30±2.23%	—
**119**	—	—	—	13.96±3.75%	1.65±0.95%	—	13.02±3.75%	1.65±0.95%	—
**184**	0.69±3.22%	—	—	14.16±5.04%	4.25±1.58%	—	14.44±5.04%	4.25±2.20%	—
**276**	—	—	—	14.46±2.10%	6.84±1.35%	1.31±1.17%	11.99±4.03%	5.29±1.54%	1.49±0.78%
**393**	—	—	—	16.87±2.12%	8.36±2.13%	3.79±0.52%	14.36±4.40%	8.36±2.13%	4.53±0.49%
**546**	1.68±3.54%	—	—	22.04±4.47%	11.17±5.16%	4.73±0.64%	22.09±4.47%	8.94±7.15%	4.72±0.64%
**897**	4.19±1.72%	—	—	32.49±1.87%	15.49±3.26%	6.94±3.37%	27.09±6.49%	13.94±4.47%	7.71±2.78%
**1315**	9.12±1.59%	—	—	43.97±2.76%	15.20±6.11%	3.07±1.39%	33.98±9.98%	17.91±4.97%	4.31±2.79%

## Discussion

### Inorganic carbon acquisition

Dinoflagellates are abundant and ecologically important in marine ecosystems, and morphologically and physiologically diverse [45]. They are also the only oxygenic photoautotrophs with type II Rubisco, the enzyme with the lowest affinity for CO_2_ among eukaryotic phytoplankton. Thus, dinoflagellates are at a disadvantage with regard to photosynthetic carbon fixation under the present ocean conditions of low CO_2_, and high O_2_ [6]. Consequently, dinoflagellates probably require an efficient CCM to compete with other phytoplankton that have higher photosynthetic and growth rates. It is a common notion that the ability of algae to raise the final pH of the medium to higher than 9.0 is an indicator of HCO_3_^-^ utilized by the species [46, 47]. The pH-drift experiments conducted in our study indicated that the final pH value in the medium of *K*. *mikimotoi* was 9.8±0.1, suggesting that *K*. *mikimotoi* could use HCO_3_^-^ in seawater. Although most phytoplankton species possess CCMs, large differences exist in their efficiencies. Owing to their rather inefficient CCMs (strongly dependent on CO_2_ as inorganic carbon source), the photosynthetic carbon fixation of the coccolithophorid *Emiliania huxleyi* and the raphidophyceae *Heterosigma akashiwo* are well below saturation at present CO_2_ levels, and therefore are more CO_2_-sensitive than species with highly efficient CCMs (which rely heavily on HCO_3_^-^ as inorganic carbon source), such as *Skeletonema costatum* and *Phaeocystis globose* [32, 48]. In the present study, Rubisco activity of *K*. *mikimotoi* did not change between 390 and 1000 ppmv, and ETR values only increase slightly (~10%), which is strong evidence that *K*. *mikimotoi*, similar to *S*. *costatum* and *P*. *globose*, relies heavily on HCO_3_^-^ as an inorganic carbon source.

CA_ext_, which catalyzes the dehydration of HCO_3_^-^ to CO_2_ at the cell surface [47–49], was found to decrease at high *p*CO_2_ conditions in other species such as *Phaeocystis globosa* and *S*. *costatum*, underlining the important role of CA_ext_ in inorganic carbon acquisition [48]. In the present study, such a HCO_3_^-^ dehydration mechanism was likely to contribute very little to the inorganic carbon acquisition in *K*. *mikimotoi*, because the activities of CA_ext_ were very low in all treatments (Fig 7). Furthermore, the minor role of CA_ext_ was also indicated by the addition of membrane impermeable CA inhibitor AZ, which only slightly inhibited the rETR of *K*. *mikimotoi* (Fig 4 and Table 4). The low CA_ext_ activity observed in *K*. *mikimotoi* was consistent with most other tested dinoflagellate species. Rost et al. [50] investigated CA_ext_ activities in *Prorocentrum minimum*, *Heterocapsa triquetra* and *Ceratium lineatum*, and found relatively low or negligible activities in all three species. Eberlein et al. [51] showed that CA_ext_ activities of the dinoflagellate *Alexandrium tamarense* acclimated to a range of *p*CO_2_ from 180 to 1200 μatm were close to detection limits, and thus only played a minor role. However, low CA_ext_ activity is not universal in all dinoflagellate species. High activity of CA_ext_ was found in *Scrippsiella trochoidea*, probably to convert the effluxing CO_2_ to HCO_3_^-^, and then utilized via the HCO_3_^-^ transporter by the cells [51]. This ‘CO_2_ recirculation mechanism’ might be especially beneficial for species with high dark respiration rates.

Furthermore, direct HCO_3_^-^ uptake via the anion-exchange (AE) protein has also been observed in other species, which suggests that HCO_3_^-^ utilization could be inhibited by the AE protein inhibitor, DIDS [52–53]. From our results, such a direct HCO_3_^-^ uptake was likely to be present in *K*. *mikimotoi*, and it was significantly reduced when the cells acclimated to 1000 and 2000 *ppmv p*CO_2_, because the rETR of *K*. *mikimotoi* was drastically depressed by the addition of DIDS, and the inhibition decreased at high *p*CO_2_ as compared with the control (Fig 4 and Table 4). Down-regulated CCMs at high *p*CO_2_ have also been found in widely distributed species such as *Skeletonema costatum*, *Emiliania huxleyi*, *Thalassionema nitzschioides* and *Pseudo-nitzschia multiseries* [48,54,55]. This down-regulation might result from the increasing diffusive CO_2_ uptake at high *p*CO_2_ conditions, since CO_2_ uptake is considered to be less energetically costly than HCO_3_^-^ uptake. Consequently, cells can optimize their allocation of energy and apportion more energy for photosynthetic carbon fixation. Marine phytoplankton productivity based on energy or carbon content might thus increase under typically resource-limited conditions in the ocean. From this point of view, species with regulated CCMs, as shown for *K*. *mikimotoi*, might have a competitive advantage in the future compared to species that do not react to high *p*CO_2_ such as *Phaeocystis globosa*, *Thalassiosira pseudonana*, *Eucampia zodiacus* and *Nitzschia navis-varingica* [48,54,55]. As a consequence, these different responses of CCMs to elevated *p*CO_2_ might change the fitness of the different group and possibly alter the distribution and succession of marine algae in natural systems.

### Growth, photosynthesis, respiration and photosynthetic electron transport

Growth is a comprehensive parameter integrating all physiological processes in marine phytoplankton, and different responses of growth to increased *p*CO_2_ have been reported among different marine phytoplankton species, with positive, negative and no significant responses. According to our experimental results, the growth rate of *K*. *mikimotoi* was not significantly affected under 1000 ppmv *p*CO_2_ conditions, while the stimulated growth rate was observed under 2000 ppmv *p*CO_2_ conditions. Enhanced growth and photosynthesis by elevated *p*CO_2_ have been reported in species such as the diatoms *Navicula pelliculosa* [56] and *Phaeodactylum tricornutum* [9], the raphidophyceae *Heterosigma akashiwo* [33] and the chlorophyte *Ulva rigida* [57]. However, other studies found no significant effects [32, 58, 59], or even negative effects [22, 60, 61] on the growth, photosynthesis or primary productivity of marine phytoplankton. With regard to the species with highly efficient and strongly regulated CCMs, stimulated growth by elevated *p*CO_2_ is generally attributed to decreased energetic cost of CCMs and HCO_3_^-^ uptake, with the saved energy allocated to support growth [62]. For *K*. *mikimotoi*, the operation of CCMs were downregulated under both 1000 and 2000 ppmv *p*CO_2_ conditions, but the saved energy from the downregulated CCMs did not stimulate growth under 1000 ppmv *p*CO_2_, probably because of enhanced respiratory carbon loss. Even though gross photosynthesis of *K*. *mikimotoi* was enhanced under 1000 ppmv *p*CO_2_ (Fig 3B), net photosynthesis was not significantly affected (Fig 3A), which may be largely caused by the enhanced dark respiration. Consequently, the growth of *K*. *mikimotoi* grown in 1000 ppmv *p*CO_2_ was not significantly stimulated, similarly to what was reported for the diatom *Thalassiosira pseudonana* [10]. When the cells acclimated to 2000 ppmv *p*CO_2_, photosynthesis of *K*. *mikimotoi* significantly increased. The explanation might be that energy was saved from down-regulation of CCM_S_, and thus resulted in increased growth, Results in the present study suggest that the responses of marine phytoplankton to future CO_2_-driven seawater acidification are not only determined by the efficiency and regulation of CCMs, but also controlled by the balance of the positive and negative effects associated with increased *p*CO_2_ and seawater acidity.

Enhanced dark respiration rates by elevated *p*CO_2_ have been reported in other species such as the diatom *Thalassiosira pseudonana*, the dinoflagellate *Alexandrium tamarense* and the diatom *Phaeodactylum tricornutum* [9, 10, 52], but not in the dinoflagellate *Scrippsiella trochoidea* and the rhodophyte *Porphyra leucosticte* [51, 63]. Conversely, a decrease in dark respiration was observed in the chlorophyte *Ulva rigida* and the diatom *T*. *pseudonana* [64, 65]. Stimulation of dark respiration under high *p*CO_2_ condition could reflect higher energy requirement due to either enhanced biosynthesis in response to increased carbon fixation, more energy demand to counteract external pH reduction and to maintain intracellular acid-base stability [66], or pH-dependent changes in the function of respiratory enzymes and altered proton gradient across the mitochondrial membrane [67]. Alternatively, decreased dark respiration by elevated *p*CO_2_ has been ascribed to the down-regulation of CCMs in order to prevent oxidative damage from excess energy [65].

Comparing the parameters derived from rapid light curves (RLCs) and induction curves, elevated *p*CO_2_ appeared to have a positive impact on the efficiency of PSII, indicated by stimulated α, Yield and decreased E_k_ at high *p*CO_2_. Generally, photosynthetic efficiency (α) represents the energetic costs of photosynthesis. Accordingly, the present study indicated that future increased *p*CO_2_ reduces the costs of photosynthesis in *K*. *mikimotoi*. Fu et al. (2007) suggested that a stimulated α at high *p*CO_2_ is attributed to the decreased energetic cost of CCMs and more efficient light use [32]. This suggestion is supported by our findings because the lower contribution of HCO_3_^-^ to inorganic acquisition was also observed in *K*. *mikimotoi*. The E_k_ (rETR_max_ / α) represents the optimum light of the photosynthetic apparatus to maintain a balance between photosynthetic energy capture and the capacity to process this energy [68]. For *K*. *mikimotoi*, an increase in α, and no significant changes in ETR_max_ at high *p*CO_2_ resulted in a decrease in the light saturation point (E_k_). This indicates that elevated *p*CO_2_ can stimulate the efficiency of light harvesting and processing of PSII, and thus fewer photons are demanded to reach the E_k_ at high *p*CO_2_. Furthermore, the lower E_k_ of *K*. *mikimotoi* in future CO_2_-induced ocean acidification also suggests that light is less likely to be a limiting factor, thus making it more competitive in light-limited conditions. By contrast, the lower E_k_ also indicated that high *p*CO_2_ lowered the light threshold at which light became excessive in *K*. *mikimotoi*, and thus the cells easily became photo-inhibited at high light conditions, an inference supported by the increased NPQ (Fig 5) and β (Table 3) at high *p*CO_2_ conditions. The operation of CCMs serves as the pathway for alleviating photo-damage through the dissipation of excess light energy [69, 70]. For *K*. *mikimotoi*, the more active CCMs in the control would consume more energy and drain more hydrogen ion out of the thylakoid lumen to the stroma, which results in a lower NPQ (Fig 5). Therefore, elevated *p*CO_2_ diminished the energy-dissipation via the down-regulated CCMs, leading to increased NPQ (Fig 5) and enhancing photo-inhibition (Table 3) at high light conditions. Wu et al. (2010) also found that elevated *p*CO_2_ enhanced the photo-inhibition of rETR in *Pheodactylum tricornutum* when exposed to high PAR, but the NPQ decreased at high *p*CO_2_ [9]. In contrast, *Thalsassiosira pseudonana* acclimated to 390 and 1000 ppmv CO_2_ showed identical photo-inhibition and NPQ when exposed to high PAR, indicating that high light tolerance was not altered by high *p*CO_2_ [10]. Such different responses among *K*. *mikimotoi*, *P*. *tricornutum* and *T*. *pseudonana* suggest that species-specific metabolic pathways might be involved in coping with elevated *p*CO_2_ and high light stress.

### Elemental composition

The elemental composition of marine phytoplankton differs intra and interspecifically. It has been hypothesized that elevated *p*CO_2_ could increase carbon assimilation, and thereby alter the elemental composition of marine phytoplankton. In this study, the similar activities of Rubisco (Fig 3D) and growth rate (Fig 1) under 390 and 1000 ppmv CO_2_ treatments suggest that photosynthetic carbon fixation did not differ between these conditions, which could explain why the total cellular carbon, nitrogen and phosphorus contents of *K*. *mikimotoi* were unaffected by the 1000 ppmv CO_2_ conditions. However, the total cellular carbon and phosphorus contents of *K*. *mikimotoi* increased under 2000 ppmv CO_2_, likely due to either enhancement of photosynthetic carbon fixation and elevated uptake of PO_4_^3-^, or to increased activities of phosphatase. Furthermore, enhancement of protein and carbohydrate synthesis also likely contributed to the increased cellular C and P. The unchanged cellular nitrogen contents under 2000 ppmv CO_2_ were probably determined by the balance between enhanced nitrogen accumulation and nitrogen loss.

### Ecological and environmental implications

It has been proposed that the dominance of bloom-forming species might be dependent on their ability to operate a regulated and efficient CCM [48,54]. In the current study, *K*. *mikimotoi* was found to have an efficient CCM, and the operation of CCM was down-regulated at high *p*CO_2_ (1000 ppmv and 2000 ppmv) conditions. However, the growth of *K*. *mikimotoi* in 1000 ppmv *p*CO_2_ was not stimulated by the reduced energetic costs of the CCM, probably due to additional carbon loss caused by enhanced dark respiration. Gao et al. [71] conducted an experiment to investigate the responses of natural phytoplankton assemblages in the South China Sea grown, over a range of light, to elevated *p*CO_2_. The results showed that growth rates of three diatom species (*Thalassiosira pseudonana*, *Phaeodactylum tricornutum*, and *Skeletonema costatum*) under 1000 ppmv *p*CO_2_ were significantly stimulated at low light levels. The different responses between the dinoflagellate *K*. *mikimotoi* and the diatoms *T*. *pseudonana*, *P*. *tricornutum* and *S*. *costatum* suggest that ongoing CO_2_-related changes could affect their dominance and succession in the future, possibly not favoring *K*. *mikimotoi* in inter-specific competitions.

Global changes not only involve increasing CO_2_ levels, but also other environmental factors such as shifts in light availability, nutrient supplies and temperature. Further studies will need to investigate whether species response to elevated *p*CO_2_ might be modulated by other interactive environmental factors. It will be critical to establish the environmental, ecological and economic consequences of *K*. *mikimotoi* blooms in a future changing ocean.
